# Age-dependent effects on radiation-induced carcinogenesis in the rat thyroid

**DOI:** 10.1038/s41598-021-98481-z

**Published:** 2021-09-27

**Authors:** Mutsumi Matsuu-Matsuyama, Kazuko Shichijo, Katsuya Matsuda, Nariaki Fujimoto, Hisayoshi Kondo, Shiro Miura, Tomomi Kurashige, Yuji Nagayama, Masahiro Nakashima

**Affiliations:** 1 Tissue and Histopathology Section, Atomic Bomb Disease Institute, Nagasaki University, 1-12-4 Sakamoto, Nagasaki, 852-8523 Japan; 2 Department of Tumor and Diagnostic Pathology, Atomic Bomb Disease Institute, Nagasaki University, 1-12-4 Sakamoto, Nagasaki, 852-8523 Japan; 3 Department of Disease Model, Research Institute for Radiation Biology and Medicine, Hiroshima University, 1-2-3 Kasumi, Minami-ku, Hiroshima, 734-8553 Japan; 4 Biostatistics Section, Atomic Bomb Disease Institute, Nagasaki University, 1-12-4 Sakamoto, Nagasaki, 852-8523 Japan; 5 National Hospital Organization Nagasaki Medical Center, 2-1001-1 Kubara, Ōmura, Nagasaki 856-8562 Japan; 6 Department of Molecular Medicine, Atomic Bomb Disease Institute, Nagasaki University, 1-12-4 Sakamoto, Nagasaki, 852-8523 Japan

**Keywords:** Biological techniques, Cancer, Endocrine cancer

## Abstract

Childhood radiation exposure is a known thyroid cancer risk factor. This study evaluated the effects of age on radiation-induced thyroid carcinogenesis in rats irradiated with 8 Gy X-rays. We analyzed cell proliferation, cell death, DNA damage response, and autophagy-related markers in 4-week-old (4W) and 7-month-old (7M) rats and the incidence of thyroid tumors in 4W, 4-month-old (4M), and 7M rats 18 months after irradiation. Cell death and DNA damage response were increased in 4W rats compared to those in controls at 1 month post-irradiation. More Ki-67-positive cells were observed in 4W rats at 12 months post-irradiation. Thyroid tumors were confirmed in 61.9% (13/21), 63.6% (7/11), and 33.3% (2/6) of irradiated 4W, 4M, and 7M rats, respectively, compared to 0%, 14.3% (1/7), and 16.7% (1/6) in the respective nonirradiated controls. There were 29, 9, and 2 tumors in irradiated 4W, 4M, and 7M rats, respectively. The expression of several autophagy components was downregulated in the area surrounding radiation-induced thyroid carcinomas in 4W and 7M rats. LC3 and p62 expression levels decreased in radiation-induced follicular carcinoma in 4W rats. Radiosensitive cells causing thyroid tumors may be more prevalent in young rats, and abrogation of autophagy may be associated with radiation-induced thyroid carcinogenesis.

## Introduction

The thyroid gland is one of the highly sensitive organs to radiation. In particular, irradiation during childhood is a known risk factor for thyroid cancer. Studies of patients with radiation therapy, survivors of atomic bomb in Japan and residents around Chernobyl nuclear reactor accident have reported that high risks of thyroid cancer is associated with radiation exposure in childhood^1–3^. Although it is epidemiologically evident that the risk of thyroid cancer following childhood exposure to radiation persists for more than 50 years after irradiation^4^, the mechanism of how childhood radiation exposure leads to a high risk of thyroid cancer remains unknown.

In a previous study, more radiation-induced thyroid tumors reportedly developed in infant (10-day-old) rats than in adult rats^5^. Lee et al. reported that the incidence of thyroid carcinoma increased with dose in 6-week-old female rats irradiated with X-rays up to 1060 rad^6^. Moreover, the thyroids of neonatal rats (1-week-old) are more sensitive to ionizing radiation at 12 Gy compared to those from adult rats, as evidenced by body weight loss and decreased colloid size in thyroid follicles^7^. However, few studies have experimentally investigated radiation-induced thyroid carcinogenesis and age effect. Meanwhile, the differences in chronic carcinogenesis following irradiation between immature and adult rats remain unclear.

Irradiation causes DNA double-strand breaks (DSBs) in thyroid cells and subsequently activates ataxia telangiectasia mutated (ATM)^8^. ATM regulates downstream proteins, including the tumor suppressor protein p53, which induces cell cycle arrest, DNA repair, apoptosis, and senescence, primarily through the transactivation of its downstream target genes^9–11^. The ATM-dependent phosphorylation of p53 Ser15 (phospho-p53^Ser15^) causes it to localize at sites of radiation-induced DNA damage, mediating the cell cycle and DNA repair to prevent carcinogenesis through chromatin-based interactions between ATM and p53^12^. Additionally, p53-binding protein-1 (53BP1), which is a DNA damage response (DDR) molecule, rapidly localizes and accumulates at the sites of DSBs, forming nuclear foci (NF). Therefore, detection of 53BP1 NF formation in a cell through immunofluorescence analysis can be considered a cytologic marker of DSBs^13,14^.

Autophagy is a homeostatic and conserved process involving lysosome-mediated degradation of cellular organelles and proteins during starvation or metabolic stress^15^. In cancer, autophagy contributes both a tumor suppressor function by preventing accumulation of damaged proteins and intracellular organelles, and a cell survival function that promotes the growth of established tumors. Although the role of autophagy in thyroid carcinogenesis has not been clarified, autophagy is involved in several steps of thyroid tumor initiation and progression^16,17^. For instance, Lin et al. reported that suppression of autophagy in human thyroid papillary carcinoma cells promotes resistance to radiation and anticancer drugs. They further suggested that the use autophagy agonists may be effective in treating refractory papillary thyroid cancer^18^. LC3 is an autophagy marker that is converted from LC3-I to LC3-II for autophagosome formation^19^ and p62 serves as an autophagic substrate^20^. We have previously reported the age-dependent effects on autophagy-related protein expression in rat thyroid glands after 8 Gy X-ray irradiation until 72 h^21^. Our previous study demonstrated that the expression of LC3-II/LC3-I and p62 proteins, and of certain autophagy-related genes, namely *Atg16l2*, *Atg9a*, *Ctss*, and *Irgm*, was higher in the thyroids of 4-week-old (4W) irradiated rats than in the thyroids of 8-month-old (8M) irradiated rats^21^. These results support the suggestion that irradiation induces autophagic activity in the thyroid glands of immature rather than adult rats.

This study aimed to determine the age-dependent effects on radiation-induced thyroid carcinogenesis molecular pathologically in vivo in rats. In this study, we evaluated changes in morphology, serum thyroid hormone levels, proliferation marker levels (Ki-67), cell death (TUNEL assay), DDR activity (53BP1 NF formation and phospho-p53^Ser15^), senescence-related molecule (p16), and autophagy-related protein (LC3-II/LC3-I and p62) in the thyroid tissues of 4W and 7M rats using immunohistochemistry and western blotting at 1, 6, and 12 months after 8 Gy X-ray irradiation. Furthermore, we quantified the incidence of thyroid tumors in 4W, 4M, and 7M rats 18 months after irradiation. The 4W, 4M and 7M rats were used as immature, young adult and adult rats respectively. Finally, we determined the differences in the cell proliferation rate, autophagy-related gene expression, and LC3 and p62 protein expression in radiation-induced thyroid carcinoma tissues between 4W and 7M rats following irradiation via immunohistochemistry, immunofluorescence, and RT^2^ profiler polymerase chain reaction (PCR) arrays. The experimental design of this study is shown in Supplementary Fig. S1.

## Results

### Morphological changes in the thyroid tissues of 4W and 7M rats after irradiation

**Figure 1:**
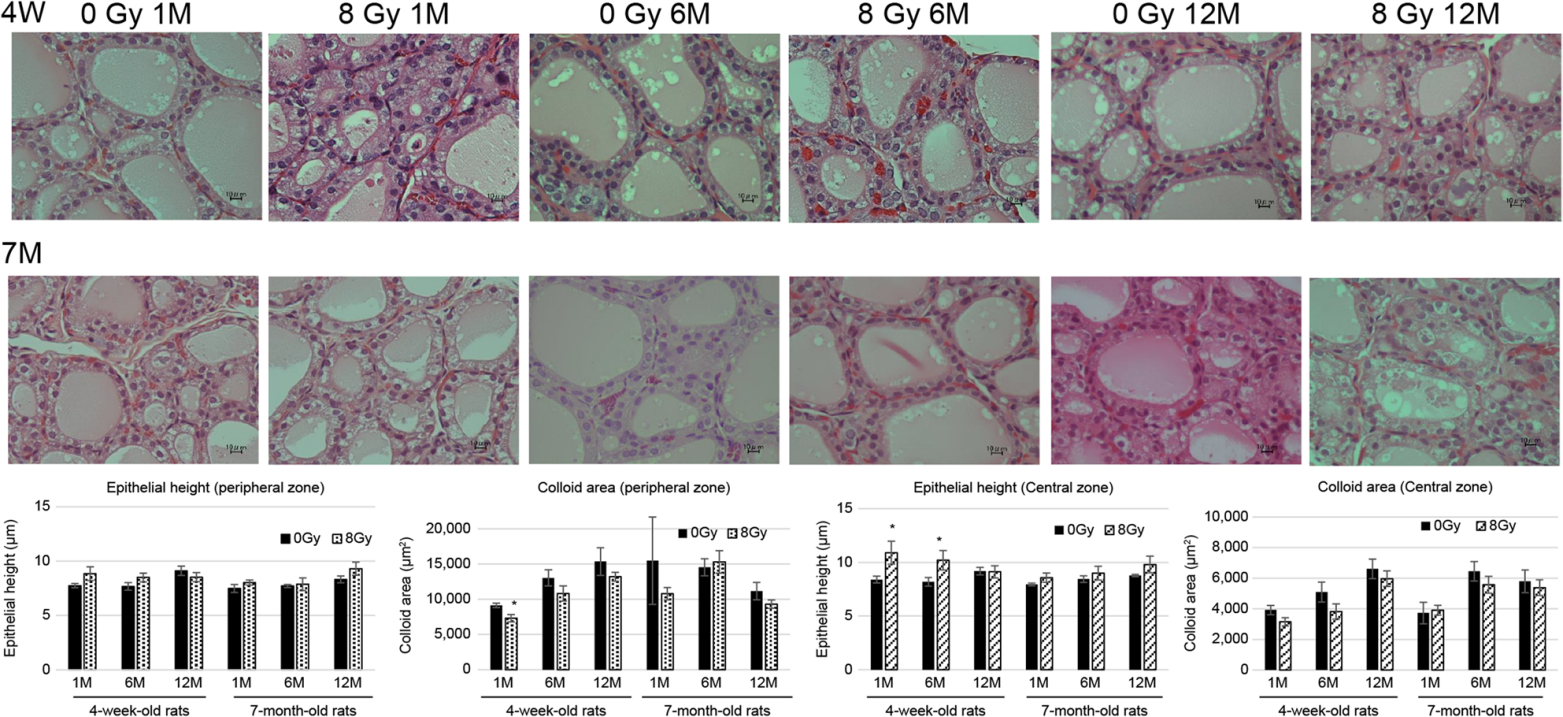
H&E staining of the thyroid central zone in nonirradiated (control) and irradiated 4W and 7M rats at 1, 6, and 12 months after irradiation (×400 magnification). Epithelial heights and colloid areas of thyroid follicles in the peripheral and central zones were measured. Data are expressed as mean ± SEM of four to six rats per data point. *p < 0.05 vs. nonirradiated group.

To evaluate morphological changes in the thyroid after irradiation, changes in epithelial height and the colloid area of the peripheral and central zones were measured in 4W and 7M rats (Fig. 1). In 4W rats, the colloid area of the peripheral zone was significantly smaller at 1 month after irradiation than in nonirradiated rats. The epithelial height of the central zone in 4W rats was significantly higher than in nonirradiated rats at 1 and 6 months after irradiation. However, there were no significant differences in the epithelial heights of the peripheral zone and colloid areas of the central zone between nonirradiated and irradiated rats at each time point. In 7M rats, there was no significant difference in the epithelial height and colloid area of the peripheral and central zones between nonirradiated and irradiated rats after irradiation. No thyroid tumor was induced in 4W and 7M rats until 12 months after irradiation.

To evaluate functional alterations in the thyroid after irradiation, the levels of thyroid hormones in the serum of 4W and 7M rats were measured 6 and 12 months after irradiation (Supplementary Table S1). TSH levels were slightly elevated in irradiated 4W rats at 6 months compared to those in nonirradiated rats; however, this change was not significant (*p* = 0.0782). There was no significant difference in TSH levels between irradiated and nonirradiated 7M rats at 6 months, between irradiated and nonirradiated 4W rats at 12 months, and between irradiated and nonirradiated 7M rats at 12 months. There was no difference in total T3 and T4 levels between irradiated and nonirradiated rats in either age group at any time point.

**Figure 2:**
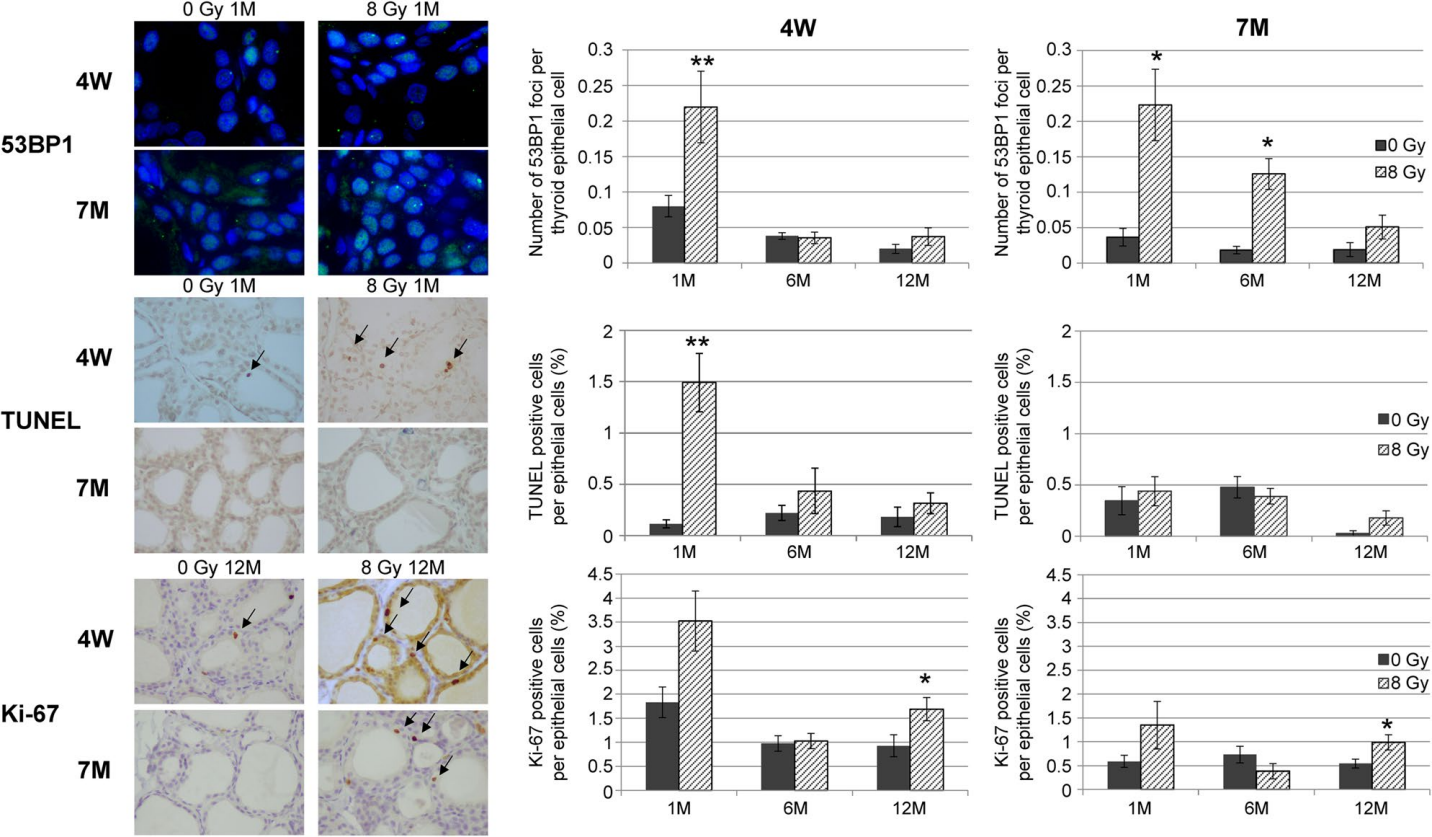
Changes in 53BP1 foci and TUNEL- and Ki-67-positive cells in nonirradiated (control) and irradiated 4W and 7M thyroid follicular epithelial cells at 1, 6, and 12 months after irradiation. Data are expressed as mean ± SEM of four to six rats per data point. *p < 0.05 and **p < 0.01 vs. nonirradiated group.

Electron microscope images of 4W and 7M rats at 1, 6, and 12 months after irradiation revealed some dead cells with nuclei and dissociated cytoplasm with vacuolation in the colloid area in 4W rats after 1 month (Supplementary Fig. S2). These dead cells were TUNEL-positive (Fig. 2); however, the dead cells that did not show cytoplasmic condensation, chromatin condensation, or formation of apoptotic bodies differed from those that underwent apoptosis^22^. Expansion of the endoplasmic reticulum and accumulation of cellular fluid were observed in the cytoplasm 1 month after irradiation in 4W rats. After 6 months, several autophagosomes were observed in both age groups. After 12 months, considerable cellular fluid accumulation was observed—particularly in 4W rats—as well as the presence of autophagosomes and mitochondrial swelling.

### Changes in 53BP1 nuclear foci, TUNEL staining, and Ki-67 expression after irradiation of 4W and 7M rats

To examine the DNA damage response, cell death, and proliferation after irradiation, changes in the number of 53BP1 nuclear foci and TUNEL- and Ki-67-positive cells were examined (Fig. 2). After irradiation, 53BP1 nuclear foci persisted until 1 month in both age groups. The nuclear foci decreased to nonirradiated levels at 4W; however, they were consistently higher in 7M rats than in the control until 6 months after irradiation before decreasing at 12 months. The number of TUNEL-positive cells in 4W rats was significantly higher than in control rats at 1 month post-irradiation, and the number of TUNEL-positive cells decreased at 6 and 12 months after irradiation. In 7M rats, there was no difference between the nonirradiated and irradiated groups at each time point. Furthermore, more Ki-67-positive cells were observed in 4W rats 1 month after irradiation compared to that in the control group; however, the difference was not significant (*p* = 0.078). Ki-67-positive cell count decreased to control levels at 6 months post-irradiation, but subsequently increased significantly at 12 months. Although the same trend was observed in 7M rats, the percentage of Ki-67-positive cells was higher in 4W than in 7M rats. This suggests that the thyroid gland had increased proliferative activity in both age groups 12 months after irradiation.

### Changes in phospho-p53^Ser15^, p16, LC3-II/LC3-I, and p62 expression in 4W and 7M rats after irradiation

**Figure 3:**
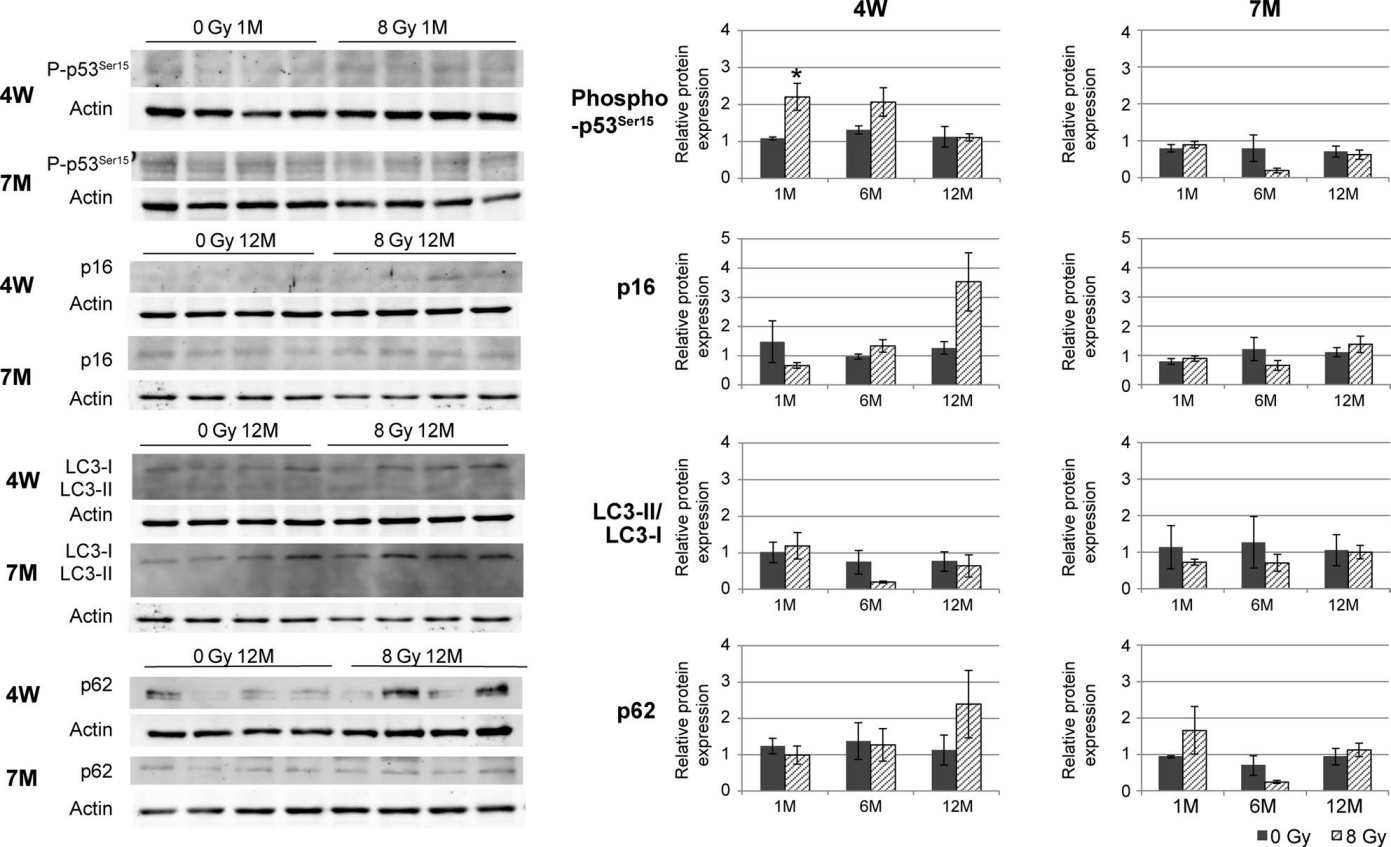
Levels of phospho-p53^Ser15^, p16, LC3-II/LC3-I, and p62 in the thyroid tissue of nonirradiated (control) and irradiated 4W and 7M rats, determined via western blotting at 1, 6, and 12 months after irradiation. Data are expressed as mean ± SEM of four to six rats per data point. *p < 0.05 vs. nonirradiated group.

To examine changes in markers for DNA damage response, senescence, and autophagy, the levels of phospho-p53^Ser15^, p16, LC3-II/LC3-I, and p62 were examined using western blotting (Fig. 3). Full-lengths blots are presented in Supplementary Figs. S3 and S4. Significantly higher levels of phospho-p53^Ser15^ were observed 1 month post-irradiation in 4W rats than in controls, while there was no change between nonirradiated and irradiated 7M rats. The levels of p16 were slightly higher in 4W rats than in control rats at 12 months post-irradiation; however, the difference was not significant. There was no significant change in the levels of LC3-II/LC3-I and p62 after irradiation in either age group.

### Incidence of radiation-induced and spontaneous thyroid tumors in 4W, 4M, and 7M rats

**Figure 4:**
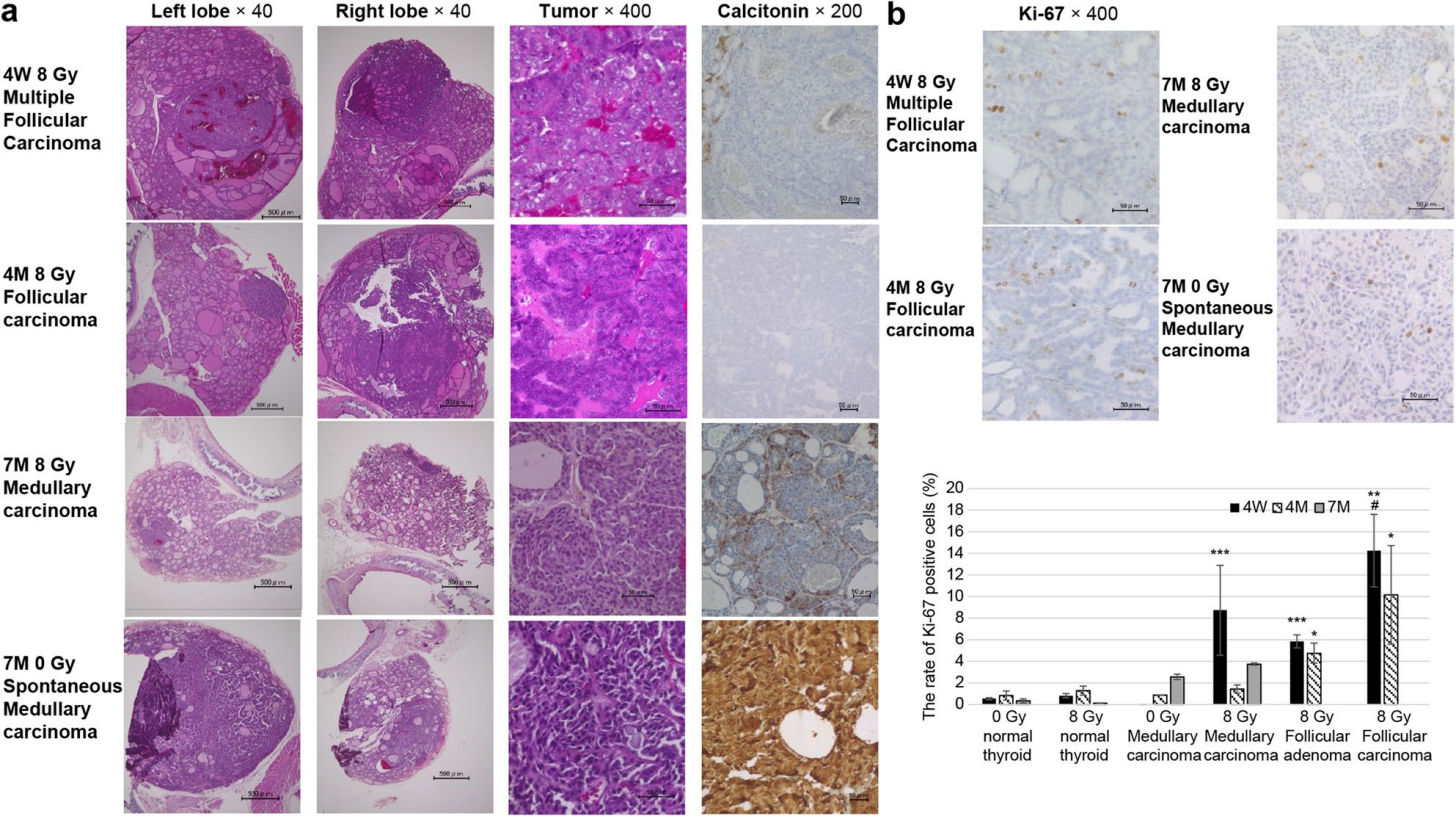
Incidence of radiation-induced and spontaneous thyroid tumors in 4W, 4M, and 7M rats. (**a**) H&E and calcitonin staining of radiation-induced follicular carcinoma in irradiated 4W and 4M rats, medullary carcinoma in irradiated 7M rats, and spontaneous medullary carcinoma in nonirradiated 7M rats. (**b**) Ki-67 staining of radiation-induced follicular carcinoma in irradiated 4W and 4M rats, medullary carcinoma in irradiated 7M rats, and spontaneous medullary carcinoma in nonirradiated 7M rats. Data are expressed as mean ± SEM of 0 Gy to normal in 4W (n = 15), 4M (n = 8), and 7M (n = 4) rats; 0 Gy to C-cell carcinoma in 4W (n = 1) and 4M (n = 2) rats; 8 Gy to normal in 4W (n = 5), 4M (n = 4), and 7M (n = 3) rats; 8 Gy to C-cell adenoma in 4W (n = 3) and 4M (n = 1) rats; C-cell carcinoma in 4W (n = 5), 4M (n = 2), and 7M (n = 2) rats; follicular adenoma in 4W (n = 8) and 4M (n = 3) rats; and follicular carcinoma in 4W (n = 3) and 4M (n = 3) rats. *p < 0.05, **p < 0.01 and ***p < 0.001 vs. 0 Gy normal. #*p* < 0.05 vs. 8 Gy follicular adenoma.

At 18 months after irradiation, thyroid tumors had developed in irradiated and nonirradiated 4W, 4M, and 7M rats. Thyroid tumors derived from C-cells were confirmed via calcitonin staining and classified as medullary carcinoma. Follicular thyroid tumors were classified according to the Boorman pathology^23^. The histological figures of radiation-induced thyroid carcinoma and spontaneous thyroid carcinoma in 4W, 4M, and 7M rats are shown in Fig. 4a. The incidence and number of thyroid tumors in irradiated and nonirradiated 4W, 4M, and 7M rats are shown in Table 1. Thyroid tumors were confirmed in 61.9% (13/21) of irradiated 4W rats and 0% (0/16) of 4W controls, 63.6% (7/11) of irradiated 4M rats and 14.3% (1/7) of 4M controls, and 33.3% (2/6) of irradiated 7M rats and 16.7% (1/6) of 7M controls. Though the incidences of thyroid tumors in 4W and 4M rats were equivalent and were higher than those in 7M rats, more thyroid tumors were observed in 4W rats compared to those in 4M rats. There were 29 tumors overall in 4W rats compared to nine in 4M rats and two in 7M rats. The radiation-induced tumor types in 4W rats included four cases of medullary carcinoma, four cases of follicular adenoma (three with multiple tumors), one case of follicular adenoma and follicular carcinoma in both lobes, one case of follicular carcinoma and medullary carcinoma in both lobes, one case with follicular carcinoma, follicular adenoma, and medullary carcinoma in both lobes, and two cases of follicular adenoma and medullary carcinoma in both lobes. The radiation-induced tumor types in 4M rats included two cases of medullary carcinoma, two of follicular adenoma, two of follicular carcinoma, and one case of follicular carcinoma, follicular adenoma, and medullary carcinoma in both lobes. One case of spontaneous medullary carcinoma occurred in a nonirradiated 4M rat. 7M rats included two cases of radiation-induced medullary carcinoma. One nonirradiated 7M rat developed a spontaneous case of medullary carcinoma in both lobes. The number of thyroid tumor cases in irradiated and nonirradiated 4W, 4M, and 7M rats, according to tumor type, is shown in Table 2. Nonirradiated rats did not exhibit follicular adenoma or follicular carcinoma in any age group. One case of spontaneous medullary carcinoma each occurred in 4M and 7M rats. In irradiated 4W and 4M rats, the incidences of follicular adenoma, follicular carcinoma, and medullary carcinoma were increased. However, no follicular adenoma or follicular carcinoma was induced in irradiated 7M rats.

**Table 1:** Incidence of thyroid tumors in irradiated and non-irradiated rats.

Age at irradiation	Number of rats at irradiation	Number of rats at sacrifice	Incidence of thyroid tumor (%)	Number of tumors	Number of tumors/number of rats at sacrifice
**0 Gy**					
4 weeks	27	16	0 (0%)	0	0
4 months	14	7	1 (14.3%)	1	0.14
7 months	13	6	1 (16.7%)	2	0.33
**8 Gy**					
4 weeks	33	21	13 (61.9%)***	29	1.38
4 months	14	11	7 (63.6%)*	9	0.82
7 months	12	6	2 (33.3%)	2	0.33

*p < 0.05, ***p < 0.001vs. non-irradiated 4W or 4M rats.

**Table 2:** Differential incidence of various thyroid tumors types in irradiated and non-irradiated rats.

Age at exposure	Number of rats	Medullary carcinoma	Follicular adenoma	Follicular carcinoma
**0 Gy**				
4 weeks	16	0 (0%)	0 (0%)	0 (0%)
4 months	7	1 (14.3%)	0 (0%)	0 (0%)
7 months	6	1 (16.7%)	0 (0%)	0 (0%)
**8 Gy**				
4 weeks	21	8 (38.1%)	8 (38.1%)	3 (14.3%)
4 months	11	3 (27.3%)	3 (27.3%)	3 (27.3%)
7 months	6	2 (33.3%)	0 (0%)	0 (0%)

Figure 4b shows the Ki-67 index in normal and tumor regions of nonirradiated and irradiated 4W, 4M, and 7M rats. The Ki-67 index of medullary carcinoma in irradiated 4W rats was significantly higher than in nonirradiated normal thyroid tissue. The Ki-67 index of follicular adenoma and follicular carcinoma in irradiated 4W and 4M rats was significantly higher than that in nonirradiated normal thyroid, and the Ki-67 index in follicular carcinoma in 4W rats was significantly higher than that in follicular adenoma.

Supplementary Fig. S5 shows electron micrographs of normal thyroid epithelial cells in nonirradiated and irradiated 4W and 7M rats and noncancerous regions of radiation-induced follicular carcinoma and medullary carcinoma in 4W and 7M rats at 18 months post-irradiation. Endoplasmic reticulum expansion, autophagosome reduction, and mitochondrial swelling were observed in the noncancerous regions of follicular carcinoma in 4W rats and medullary carcinoma in 7M rats. However, endoplasmic reticulum expansion, autophagosome reduction, and mitochondrial swelling were more severe in 4W rats than in 7M rats.

### Expression of autophagy-related genes in thyroid cancer cases from irradiated and nonirradiated 4W and 7M rats

To examine changes in the expression of autophagy-related genes in radiation-induced thyroid cancers from 4W and 7M rats, an autophagy-focused PCR array was performed on radiation-induced follicular carcinoma and medullary carcinoma cases from 4W rats and on radiation-induced and spontaneous medullary carcinoma cases from 7M rats (Table 3). In the noncancerous region of follicular carcinoma in 4W rats, autophagy regulatory genes including *Cdkn2a*, *Ctss*, *Cxcr4*, *Tgfb1*, *Tnf*, and *Tp53* were upregulated, whereas *Eif2ak3*, *Mapt,* and *Pim2*, were downregulated. Moreover, the expression of autophagy machinery components, such as *Gabarap* and *Map1lc3b*, decreased. In radiation-induced medullary carcinoma in 4W rats, the expression of *Cdkn2a* and *Cxcr4* increased, whereas that of seven autophagy regulatory genes (*Dapk1*, *Eif2ak3*, *Gaa*, *Hgs*, *Mapkl4*, *Mapt*, and *Pim2*) and five autophagy machinery components (*Atg4b*, *Atg5*, *Gabarapl2*, *Rab24*, and *Wipi1*) decreased. In radiation-induced medullary carcinoma in 7M rats, the expression of *Ctss* and *Cxcr4* increased, whereas the expression of seven autophagy regulatory genes (*Dapk1*, *Eif2ak3*, *Eif4g1*, *Hgs*, *Htt*, *Mapt*, and *Nfkb1*) and one autophagy machinery component (*Atg4b*) decreased, which was consistent with the decreased gene expression profile in the cancerous region of spontaneous medullary carcinoma in 7M rats. In spontaneous medullary carcinoma in 7M rats, the expression level of *Mapk8* was increased, and the expression levels of two other genes, *Ulk1* and *Wipi1*, were decreased.

**Table 3:** Expression of autophagy-related genes in radiation-induced and spontaneous thyroid carcinoma cases in 4W and 7M rats.

	4W Radiation-induced follicular carcinoma (noncancerous)		4W Radiation-induced medullary carcinoma (noncancerous)		7M Radiation-induced medullary carcinoma (noncancerous)		7M Spontaneous medullary carcinoma (cancerous)	
	Fold change	*p*-value	Fold change	*p*-value	Fold change	*p*-value	Fold change	*p*-value
**Autophagy regulation**								
*Cdkn2a*	5.58	0.012	1.79	0.032	–	–	–	–
*Ctss*	1.53	0.0075	–	–	2.04	0.063	–	–
*Cxcr4*	3.54	0.025	2	0.015	5.46	0.019	–	–
*Tgfb1*	2.05	0.0055	–	–	–	–	–	–
*Tnf*	2.51	0.020	–	–	–	–	–	–
*Tp53*	1.66	0.017	–	–	–	–	–	–
*Mapk8*	–	–	–	–	–	–	2.43	0.035
*Dapk1*	–	–	0.50	0.02	0.16	0.0078	0.2	0.0014
*Eif2ak3*	0.46	0.022	0.44	0.012	0.44	0.010	0.58	0.016
*Eif4g1*	–	–	–	–	0.39	0.020	0.46	0.010
*Gaa*	–	–	0.48	0.036	–	–	–	–
*Hgs*	–	–	0.56	0.035	0.46	0.084	0.48	0.021
*Htt*	–	–	–	–	0.29	0.025	0.4	0.013
*Mapk14*	–	–	0.47	0.054	–	–	–	–
*Mapt*	0.23	0.0035	0.40	0.0081	0.48	0.051	0.49	0.059
*Nfkb1*	–	–	–	–	0.49	0.044	0.47	0.0035
*Pim2*	0.32	0.015	0.38	0.015				
**Autophagy machinery components**								
*Atg4b*	–	–	0.49	0.036	0.51	0.030	0.65	0.0086
*Atg5*	–	–	0.49	0.047	–	–	–	–
*Gabarap*	0.54	0.033	–	–	–	–	–	–
*Gabarapl2*	–	–	0.38	0.027	–	–	–	–
*Map1lc3b*	0.43	0.075	–	–	–	–	–	–
*Rab24*	–	–	0.48	0.021	–	–	–	–
*Ulk1*	–	–	–	–	–	–	0.32	0.050
*Wipi1*	–	–	0.68	0.037	–	–	0.31	0.012

Fold change represents the average of triplicate experiments from single rats. Results are expressed relative to nonirradiated normal thyroids in 4W or 7M rats.

### LC3 and p62 expression in radiation-induced and spontaneous thyroid cancer in 4W and 7M rats by immunofluorescence

**Figure 5:**
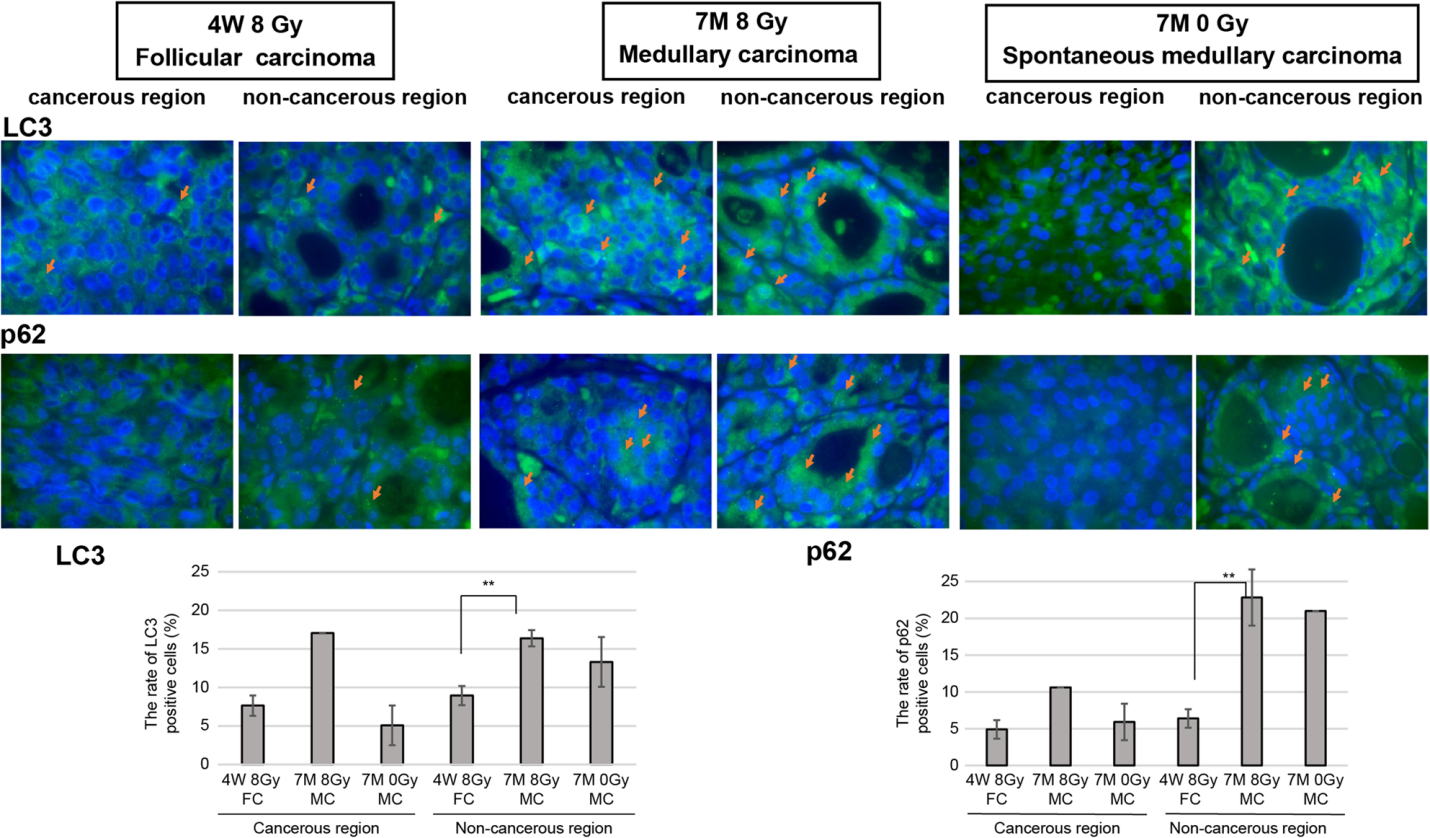
Expression of LC3 and p62 in cancerous and noncancerous regions of follicular carcinoma (FC) in irradiated 4W rats and medullary carcinoma (MC) in irradiated and nonirradiated 7M rats, as revealed by immunofluorescence (×1000 magnification). LC3- and p62-positive cells are indicated by the arrows. The graph shows the rate of LC3- and p62-positive cells. Data are expressed as mean ± SEM of four to five areas from a case except for one area of 7M 8 Gy MC (LC3 and p62) and 7M 0 Gy MC (p62). **p < 0.01.

Immunofluorescence analysis revealed that, in 4W rats, few punctated LC3 and p62 were expressed in the cytoplasm in either cancerous or noncancerous follicular carcinoma regions. However, in radiation-induced medullary carcinoma in 7M rats, LC3 and p62 expression was observed in the cancerous and noncancerous regions. There were significance differences in LC3- and p62-positive cells in noncancerous regions between radiation-induced follicular carcinoma in 4W rats and medullary carcinoma in 7M rats (*p* < 0.01). In spontaneous medullary carcinoma in 7M rats, punctated LC3 and p62 expression was observed in the noncancerous regions, but not in the cancerous regions. Hence, a decrease in autophagy may be prominent in both the cancerous and noncancerous regions of 4W rats with follicular carcinoma (Fig. 5).

## Discussion

This study molecular pathologically demonstrated the difference in radiation responses in the thyroid gland between 4W and 7M rats in the late phase following exposure to radiation. Histologically, the height of the follicular epithelial in the central zone of the irradiated thyroid gland was taller in 4W rats than in 7M rats for up to 6 months post-irradiation. The colloid area in the peripheral zone decreased significantly in 4W rats at 1 month post-irradiation. Our recent study also revealed an increase in the height of the epithelium with a decrease in colloid area of thyroid follicle after 12 Gy irradiation in neonatal rats. Serum TSH levels were significantly increased in irradiated neonatal rats. It has been suggested that epithelial height is inversely related to serum TSH levels^7^. However, in this study, the serum TSH level was only slightly increased in 4W rats compared to that in the control at 6 months post-irradiation, and this change was not significant. The morphological changes after 8 Gy irradiation in 4W rats were lesser than those after 12 Gy irradiation in the neonatal period. The change in TSH level may also be small, and we could not measure serum thyroid hormone levels at 1 month post-irradiation. Changes in serum TSH levels may occur in the first 6 months after irradiation. Immunohistochemistry for Ki-67 expression revealed an increased proliferative activity in irradiated follicular cells in 4W rats compared to that in 7W rats at both 1 month and 12 months, indicating more proliferative stress, and therefore, cellular hypersensitivity to DNA damage agents in 4W rats as a late effect following irradiation.

Regarding DDR activity, in our previous study on early phase post-irradiation, the number of 53BP1 NF and the level of phospho-p53^Ser15^ expression in the thyroid tissue peaked at 3 h after irradiation irrespective of age, gradually decreasing thereafter for up to 72 h, and no increase in apoptosis was observed in both age groups until 72 h post irradiation^21^. These results suggest that although DSBs are induced in irradiated thyroid irrespective of age, damaged follicular cells are not eliminated by apoptosis at early phase post-irradiation. In late phase in this study, an increased number of 53BP1 NF was observed in 4W and 7M rats; however, TUNEL-positive cells and phospho-p53^Ser15^ expression in thyroid tissues were observed in 4W rats but not in 7M rats compared to that in control at 1 month post-irradiation. Thus, DDR may re-activate in irradiated thyroid cells at late phase post-irradiation to eliminate damaged follicular cells in 4W rat thyroid with a higher proliferative activity at 1 month post-irradiation. Because the induction of 53BP1 NF is considered as an indicator of DSBs, the increased number of 53BP1 NF in mitotically active follicular cells may be associated with an increased predisposition to thyroid cancers through mismatch repair. Thus, our analysis demonstrated the involvement of endogenously activated DDR leading to genomic instability in young-age related radiation-induced thyroid carcinogenesis. Indeed, this study demonstrated the higher incidence of thyroid tumors exhibiting a high proliferative activity in 4W rats at 18 months post-irradiation.

Observations of the morphology via electron microscopy revealed an increase in the cytoplasmic fluid at 1 month after irradiation, and remarkable changes in the cytoplasm, including mitochondrial swelling surrounding radiation-induced thyroid carcinoma, in 4W rats. The morphology of cell death as examined via electron microscopy at 1 month post-irradiation seemed to be different from typical apoptosis found in the irradiated intestine^24^, but was similar to degenerating follicular cells in the lumen^25^. Western blot analysis for expression of senescence and autophagy markers, such as p16, LC3-II/LC3-I, and p62, did not demonstrate significantly changes until 12 months post-irradiation in either age group. Conversely, immunofluorescence analysis at 18 months post-irradiation revealed the expression of puncta LC3 and p62 proteins in irradiated medullary carcinoma in 7M rats, but their expression was decreased in the cancerous and noncancerous regions of radiation-induced follicular carcinoma in 4W rats. Furthermore, an analysis with autophagy-pathway-specific PCR array revealed the downregulations of several autophagy-related genes in the area surrounding radiation-induced thyroid carcinoma in both 4W and 7M rats. Thus, we suggest an association of deficient autophagic activity with young age-related radiation-induced thyroid carcinogenesis around cancerous phase post irradiation. It has been reported that a whole-body 2 Gy gamma radiation dose can sustainably downregulate autophagy and antioxidant signaling, while upregulating proliferative signaling and oxidant production in the mouse intestine^26^. Kurashige et al. have reported that long-term basal autophagy deficiency in *Atg5* knockout mice induces TUNEL-positive thyrocytes with excess oxidative stress, which is indicated by 8-hydroxy-2′-deoxyguanosine and 53BP1 NF. These findings demonstrate that thyrocytes gradually undergo degradation or cell death in the absence of basal levels of autophagy, indicating that autophagy is critical for the quality control of thyrocytes^27^. Because autophagy-deficient cells accumulate injured organelles, including mitochondria, and increase reactive oxygen species (ROS) levels, which seems to contribute to tumorigenesis^28^, autophagy deficiency in irradiated thyroid cells at young age may lead to failure of mitochondria function, higher ROS production, and more severe thyroid carcinoma compared to that in 7M irradiated rats.

A study of mice implanted with a human thyroid epithelial cell line (HT ori-3 cells) after irradiation with high-dose gamma radiation revealed that increased tumorigenicity and altered gene expression involved apoptosis, regulation of transcription, immune response, and receptor signaling pathways^29^. Recently, our comprehensive analysis using RNA microarray identified altered expression of more than 3000 genes in 4 Gy-irradiated nontumorous thyroid glands in 7W Wistar-Kyoto rats at 16 months; among these, droplet digital PCR revealed deficiencies in DNA damage response/repair mechanisms and cell–cell adhesion in the late phase of radiation-induced thyroid carcinogenesis. In addition, dysregulation of cell cycle-related molecules, including *Cdkn1a* and *Cdkn2a* overexpression in irradiated thyroids, can accelerate radiation-induced carcinogenesis^30^. In this study, the overexpression of genes encoding coregulators of autophagy and the cell cycle, such as *Tp53*, *Cdkn2a*, and *Tgfb1*, coregulators of autophagy and apoptosis, such as *Tnf* and *Cxcr4,* as well as genes associated with autophagy in response to other intracellular signals, such as *Ctss*, was observed in radiation-induced carcinoma in 4W rats. The expression of the genes *Map1lc3b* and *Atg5*, which are important for the formation of autophagosome, was downregulated in radiation-induced carcinoma in 4W rats. Thyroid cancers from 4W rats had high proliferative activity and large genetic changes, in addition to a decrease in the expression of genes associated with components of autophagy machinery.

In our previous study, the proportion of proliferating thyroid follicular epithelial cells was significantly higher in the 4W group (11%) than the 8M group (0.2%). After irradiation, the number of proliferating cells was significantly decreased in the 4W group, but not in the 8M group^21^. It has been reported that the high proliferative activity of thyroid cells at younger ages may correlate with a higher risk of radiation-induced thyroid cancer in children than adults; however, it has also been suggested that other mechanisms that cause a high risk of thyroid cancer in children are likely to exist^31^. Takano suggested a model in which thyroid carcinogenesis was associated with fetal cell carcinogenesis. In that model, two thyroid cancer types were proposed: mature cancer arising from differentiated fetal cells, which are thyroblasts, and immature cancer originating from undifferentiated thyroid stem cells^32^. It is suggested that the high risk of thyroid carcinogenesis in irradiated young rats may be due to the long-term survival of immature thyroid cells, but details on thyroid stem cells are limited.

In conclusion, radiation exposure in young rats induces multiple thyroid tumors, including highly proliferative thyroid cancer. The multiplicity of thyroid tumorigenesis in younger rats exposed to radiation suggested that radiosensitive cells causing thyroid tumors may be more prevalent in younger rats, and these cells might be immature thyrocytes possessing stemness because they can survive for a life-long post-irradiation. The abrogation of the autophagy pathway may be associated with radiation-induced thyroid carcinogenesis. Further studies are needed to elucidate the relationship between radiation-induced carcinogenesis in young rats and autophagy deficiency.

## Methods

### Animals and irradiation

Four-week-old (4W) immature (100–140 g, n = 98), 4-month-old (4M; 505–600 g, n = 28), and 7-month-old (7M; 555–620 g, n = 60) adult male Wistar rats were obtained from Charles River Japan (Atsugi, Japan). All animals were allowed free access to food and tap water and were housed in specific pathogen-free conditions at the Biomedical Research Center of Nagasaki University. This experimental protocol was approved by the Institutional Animal Care and Use Committee of the Biomedical Research Center of Nagasaki University (approval no. 1612211352). All methods were performed in accordance with the relevant guidelines and regulations. The study protocol adhered to the ARRIVE guidelines.

Irradiation was performed in the morning; 4W (n = 54), 4M (n = 14), and 7M (n = 30) rats received external exposure to 8 Gy X-rays on the anterior neck (2 cm × 2 cm area in 4W rats and 3 cm × 4 cm in 4M and 7M rats) using an ISOVOLT TITAN 32 X-ray (Toshiba, Tokyo, Japan) under the following conditions: 200 kV, 15 mA device with 0.5 mm aluminum + 0.5 mm copper + 5 mm aluminum filters. The dose rate was 0.5531 Gy/min. Control rats were not irradiated, but were otherwise treated similarly (4W, n = 44; 4M, n = 14; 7M, n = 30). Animals that died suddenly 6 months after irradiation were excluded from the data analysis.

After sacrificing rats via deep anesthesia, which was induced by intraperitoneal injection of pentobarbital sodium 160 mg/kg (4W, 0 Gy, n = 17; 4W, 8 Gy, n = 20; 7M, 0 Gy, n = 15; 7M, 8 Gy, n = 17), histological and western blot analyses were performed on thyroid tissues from irradiated and nonirradiated 4W and 7M rats at 1, 6, and 12 months post-irradiation. The right lobe of the thyroid tissue was immediately frozen and stored at – 80 °C for western blot analysis. The left lobe of the thyroid tissue was resected and fixed in 15% formalin neutral buffer solution (FUJIFILM Wako Pure Chemical Corporation, Osaka, Japan) overnight to prepare paraffin sections. A portion of the thyroid tissue was resected for electronic microscopy.

Thyroid tissues were removed from 4W, 4M, and 7M rats between 15 and 18 months after irradiation to analyze radiation-induced thyroid tumors. Nonirradiated control thyroid tissues were also removed from nonirradiated rats. Animals that experienced weight loss or weakness between 15 and 18 months post-irradiation were sacrificed (4W, 0 Gy, n = 3; 4W, 8 Gy, n = 4; 4M, 0 Gy, n = 2; 4M, 8 Gy, n = 4; 7M, 0 Gy, n = 3; 7M, 8 Gy, n = 2). All remaining animals were sacrificed 18 months after irradiation. The intact thyroid was removed with a portion of the trachea. The thyroid was cut in the middle, and the lower half was fixed overnight for paraffin sections. The upper half of the left and right lobes of the thyroid tissues was soaked in RNA stabilization buffer (Qiagen, Tokyo, Japan) for RNA extraction. A portion of the thyroid tissue was resected through electron microscopy.

### Thyroid hormone assessment

To assess thyroid hormone levels, blood samples were collected from the hearts of 4W and 7M rats at 6 and 12 months after irradiation, or without irradiation, under deep anesthesia, which was induced by intraperitoneal injection of an anesthetic mixture: 0.2 mg/kg of medetomidine, 3.1 mg/kg of midazolam, and 2.5 mg/kg of butorphanol. Blood samples were centrifuged at 1500×*g* for 15 min, and the serum was stored at – 80 °C. Total serum triiodothyronine (T3) and thyroxine (T4) levels were measured using an ELISA kit (Alpco Diagnostics, Salem, NH, USA), as described previously^33^. Serum TSH levels were measured by the Fujifilm Wako Shibayagi Corporation (Shibukawa, Japan).

### Histological analysis

After fixation, thyroid tissues were embedded in paraffin blocks, cut into 4-µm sections, and stained with hematoxylin and eosin (H&E). H&E-stained images were taken using an all-in-one microscope BZ-9000 (Keyence Co., Osaka, Japan). Five different areas of the peripheral and central zones were selected from each specimen at 200× magnification. The five largest thyroid follicles from each field of view were selected for measurement. The epithelial height and colloid area were measured using the BZ-II Analyzer software (Keyence Co.) as described previously^7^. The first and second layers of follicles from the outside were defined as the peripheral zone, while the other area was defined as the central zone^34^. The number of thyroid tumors in both lobes was determined 18 months after irradiation.

Thyroid tissues for electron microscopy were fixed in 2.5% glutaraldehyde solution buffer (pH 7.4; TAAB, Reading, UK) for 4 h at 4 °C. Post-fixation was performed with 2% osmium tetroxide solution buffer (pH 7.4; Merck, Darmstadt, Germany) for 2 h at 4 °C. Tissues were embedded in Epon 812. Ultrathin sections were cut using an ultramicrotome (Ultracut S, Leica, Vienna, Austria) with a diamond knife, double stained with uranyl acetate and lead nitrate, and observed under an electron microscope (JEM-1200EX, JEOL, Tokyo, Japan) at an accelerating voltage of 80 kV.

### Calcitonin and Ki-67 immunohistochemistry

Immunohistochemical staining was performed for calcitonin and Ki-67, as described previously^21^. Anti-calcitonin polyclonal (Nichirei Bioscience, Tokyo, Japan) and anti-Ki-67Monoclonal (MIB-5, Agilent, Santa Clara, CA, USA) antibodies diluted 1:50 in Dako Real™ antibody diluent (Agilent) were used. Simple Stain Rat Max-PO ® (Nichirei) or LSAB^®^2 system-HRP (Agilent) were used for calcitonin or Ki-67 staining, respectively, according to the manufacturer’s instructions. Ki-67-positive cells were counted in at least five fields per rat from four to six rats for each data point at 1, 6, and 12 months after irradiation. Images were captured using a Nikon Camera Control Unit DS-L2 (Nikon, Tokyo, Japan) (400× magnification). The Ki-67-positive cells in the normal and tumor regions (Ki-67 index) were counted under the same conditions 18 months after irradiation.

### 53BP1, LC3, and p62 immunofluorescence staining

The sections were incubated with anti-53BP1 (Bethyl Labs, Montgomery, TX, USA), anti-LC3 (Abcam, Cambridge, UK), or anti-p62/SQSTM1 (MBL, Nagoya, Japan) polyclonal antibodies at 1:200, 1:1000, or 1:1000 dilutions, respectively, at 4 °C overnight after microwave treatment in citrate buffer. Sections were incubated with Alexa Fluor 488-conjugated goat anti-rabbit antibody (Invitrogen, Carlsbad, CA, USA), counterstained with DAPI (Vector Laboratories, Burlingame, CA, USA), and photographed using a BZ-9000. The number of 53BP1 nuclear foci was determined in at least six fields per rat from four to six rats for each data point at 1, 6, and 12 months after irradiation (1000× magnification). LC3 and p62 expressing cells were counted from four to five areas of cancerous and noncancerous regions of follicular carcinoma in irradiated 4W rats and medullary carcinoma in irradiated and nonirradiated 7M rats, except for one area of cancerous region of medullary carcinoma in irradiated 7M rats and noncancerous region of medullary carcinoma in nonirradiated 7M rats (1000× magnification). The rate of positive cells was expressed as ratios of the total nuclear number in the area.

### Terminal deoxynucleotidyl transferase-mediated dUTP nick end labeling (TUNEL) staining

TUNEL staining was performed using an ApopTag peroxidase in situ apoptosis detection kit (Millipore Co., Temecula, CA, USA). The slides were stained as described previously^35^. TUNEL-positive cells were counted in at least five fields per rat from four to five rats for each data point (400× magnification) and captured using the Nikon Camera Control Unit DS-L2.

### Western blotting

Thyroid samples from 4W and 7M rats at 1, 6, and 12 months after irradiation were analyzed for phospho-p53^Ser15^, p16, LC3, and p62 expression. Nonirradiated 4W and 7M thyroid tissues (controls) were removed at the same time and frozen immediately. Total protein was extracted from the tissues^21,35^. Proteins (30 µg) were subjected to sodium dodecyl sulfate–polyacrylamide gel electrophoresis (SDS-PAGE) and transferred to nitrocellulose blotting membranes (GE Healthcare, Tokyo, Japan). Membranes were incubated with anti-p16 (Santa Cruz Biotechnology, Dallas, TX, USA), anti-phospho-p53^Ser15^ (Cell Signaling Technology, Danvers, MA, USA), anti-LC3, anti-p62/SQSTM1 (MBL), or anti-actin (Sigma-Aldrich, St. Louis, MO, USA) antibodies. This was followed by incubation with an HRP-conjugated anti-mouse IgG antibody (Invitrogen) or HRP-conjugated anti-rabbit IgG (GE Healthcare). Chemiluminescence (ECL Prime, GE Healthcare) was performed according to the manufacturer’s protocol. Protein detection was performed using LAS4000 (FUJIFILM) and quantified using NIH ImageJ software. Data are expressed as previously described^21^.

### RNA isolation, reverse transcription, and quantitative real-time PCR

Thyroid samples were obtained from four cases of carcinoma, follicular carcinoma, and medullary carcinoma from irradiated 4W rats; a medullary carcinoma from irradiated 7M rats; and a spontaneous medullary carcinoma from a nonirradiated 7M rat. Normal thyroid samples were obtained from one nonirradiated 4W and one nonirradiated 7M rat. Tissue was immersed in RNA Protect^®^ Tissue Reagent (Qiagen) for 24 h at 4 °C and stored at – 80 °C. Total RNA was isolated using the QIAzol Lysis Reagent and RNeasy Mini Kit (Qiagen). cDNA synthesis and reverse transcription were conducted using the RT^2^ First Strand Kit (Qiagen) with 1 μg total RNA. cDNA was mixed with RT^2^ SYBR Green Master Mix (Qiagen) for use in the RT^2^ Profiler PCR array (catalog no. PARN-084ZA; Qiagen), which contained 84 primer pairs of rat autophagy-related genes, as described previously^21^. Quantitative real-time PCR was carried out using a Takara TP-800 thermal cycler (Takara, Shiga, Japan) following the manufacturer’s instructions. Samples were analyzed in triplicate. Fold change was calculated using the 2^−ΔΔCt^ method (Geneglobe.Qiagen.com/jp/analyze/) as previously described^21^. Results were expressed relative to nonirradiated normal thyroid samples from 4W and 7M rats.

### Statistical analyses

All data are expressed as the mean ± standard error of the mean (SEM). Differences between nonirradiated and irradiated groups were analyzed using the Mann–Whitney U test. The incidence of thyroid tumors was examined using the chi-square test. Statistical analyses were conducted using SAS statistical software (SAS Institute Inc., Cary, NC, USA) and BellCurve for Excel software (Social Survey Research Information Co., Ltd., Tokyo, Japan). Two-tailed *p*-values < 0.05 were considered statistically significant.

## Supplementary Information

Supplementary Information.

## Data Availability

All data generated or analyzed during this study are included in this article.
