# Supplementary material for: Age-dependent effects on radiation-induced carcinogenesis in the rat thyroid
Source: Sci Rep. 2021 Sep 27;11:19096. doi: 10.1038/s41598-021-98481-z (PMC8476610; doi:10.1038/s41598-021-98481-z)
Supplement: Supplementary file 1 — Supplementary Information. [file 41598_2021_98481_MOESM1_ESM.docx]

**Supplementary Information**

**Age-dependent effects on radiation-induced carcinogenesis in the rat thyroid**

Mutsumi Matsuu-Matsuyama, Kazuko Shichijo, Katsuya Matsuda, Nariaki Fujimoto, Hisayoshi Kondo, Shiro Miura, Tomomi Kurashige, Yuji Nagayama, Masahiro Nakashima

**
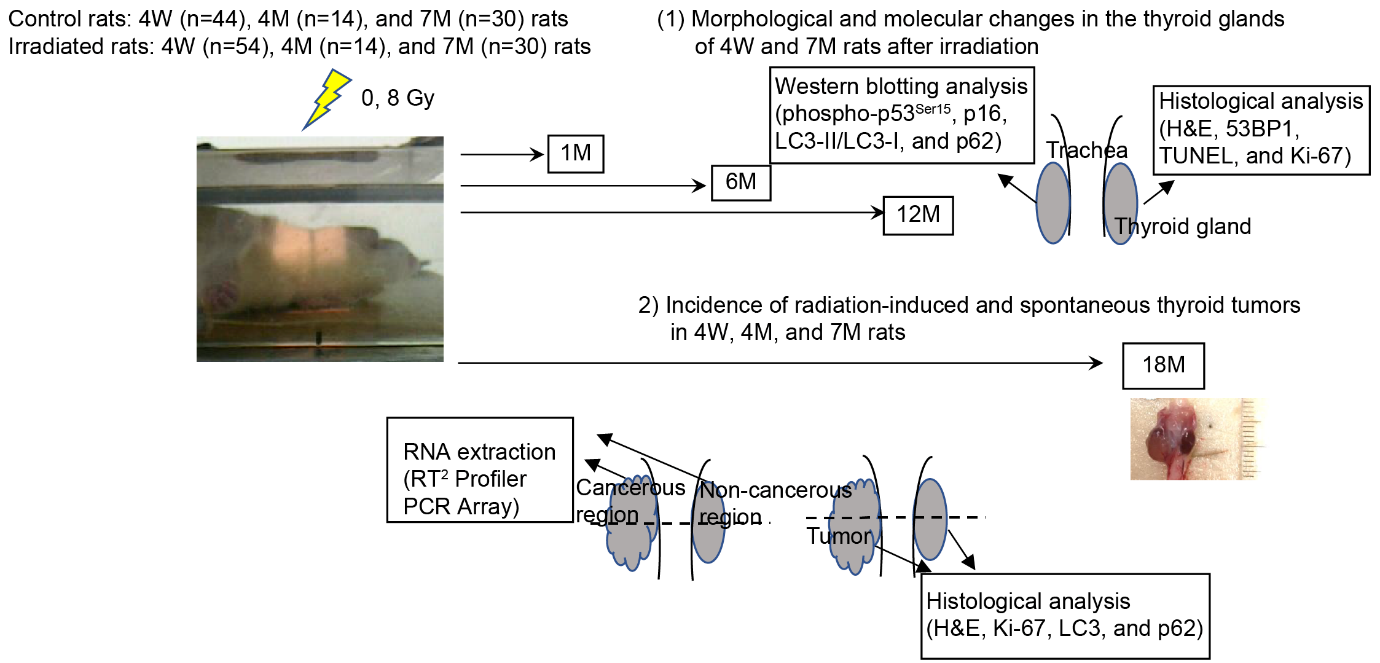
**

**Supplementary Fig. S1** Experimental design of the study. Four-week-old (4W), four-month-old (4M) and seven-month-old (7M) Wistar rats were divided into nonirradiated (control) and focal irradiated with 8 Gy of X-ray groups. Thyroid glands were used for analysis at 1, 6, 12 or 18 months after irradiation.

**Supplementary Table S1** Serum TT3, TT4 and TSH levels in rats exposed to focal X-ray irradiation

|  | Total T3 (ng/mL) | Total T4 (mg/dL) | TSH (ng/mL) |
| --- | --- | --- | --- |
| 6 months after irradiation | | | |
| 4W 0 Gy | 3.28 ± 0.40 (n = 6) | 5.43 ± 0.20 (n = 6) | 0.97 ± 0.120 (n = 6) |
| 4W 8 Gy | 3.35 ± 0.59 (n = 6) | 5.44 ± 0.69 (n = 5) | 1.27 ± 0.068 (n = 6) |
| 7M 0 Gy | 1.87 ± 0.11 (n = 5) | 3.56 ± 0.45 (n = 5) | 1.22 ± 0.280 (n = 6) |
| 7M 8 Gy | 2.04 ± 0.26 (n = 6) | 4.29 ± 0.42 (n = 6) | 2.32 ± 0.940 (n = 6) |
| 12 months after irradiation | | | |
| 4W 0 Gy | 2.27 ± 0.18 (n = 5) | 3.83 ± 0.19 (n = 5) | 1.85 ± 0.430 (n = 5) |
| 4W 8 Gy | 2.15 ± 0.21 (n = 5) | 4.31 ± 0.42 (n = 5) | 1.34 ± 0.350 (n = 7) |
| 7M 0 Gy | 2.14 ± 0.22 (n = 5) | 2.26 ± 0.20 (n = 5) | 2.19 ± 0.250 (n = 5) |
| 7M 8 Gy | 2.35 ± 0.27 (n = 4) | 2.3 ± 0.33 (n = 4) | 2.11 ± 0.150 (n = 4) |

Values presented as mean ± SEM

**
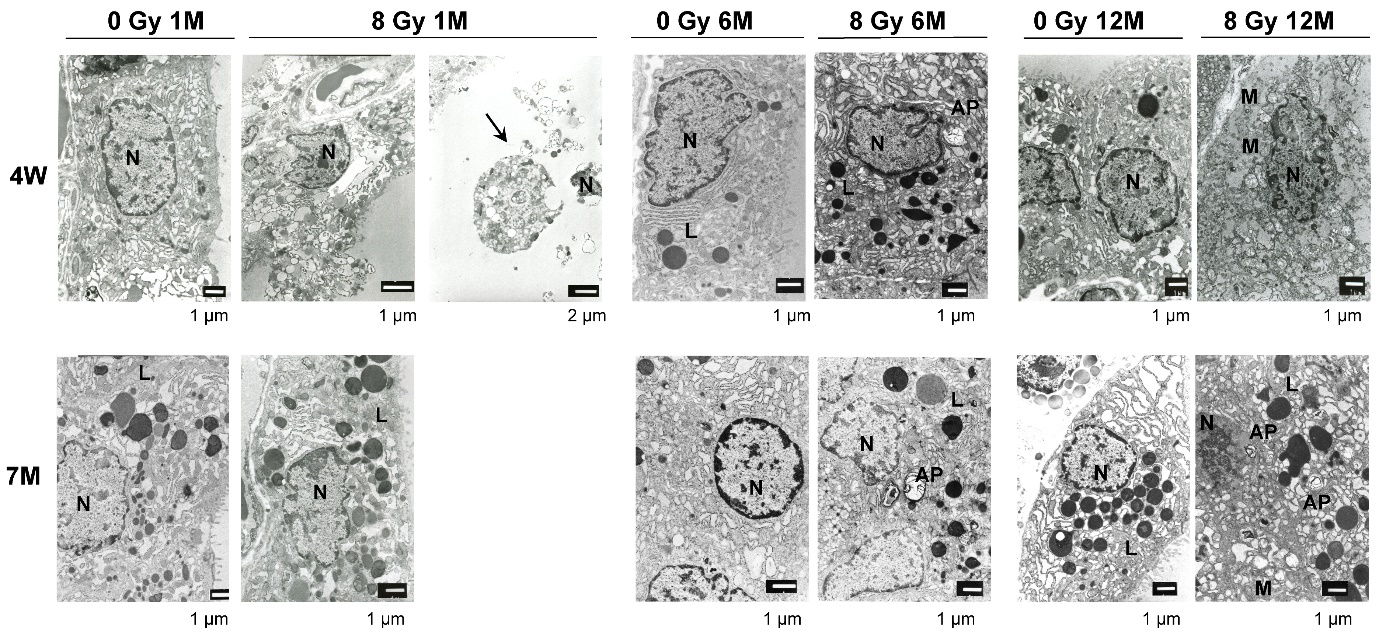
Supplementary Fig. S2** Electron microscopy of irradiated 4W and 7M thyroid follicular epithelial cells at 1, 6, and 12 months after irradiation

N, nuclear; L, lysosome; AP, autophagosome; M, mitochondria; arrow, dead cells in colloid area


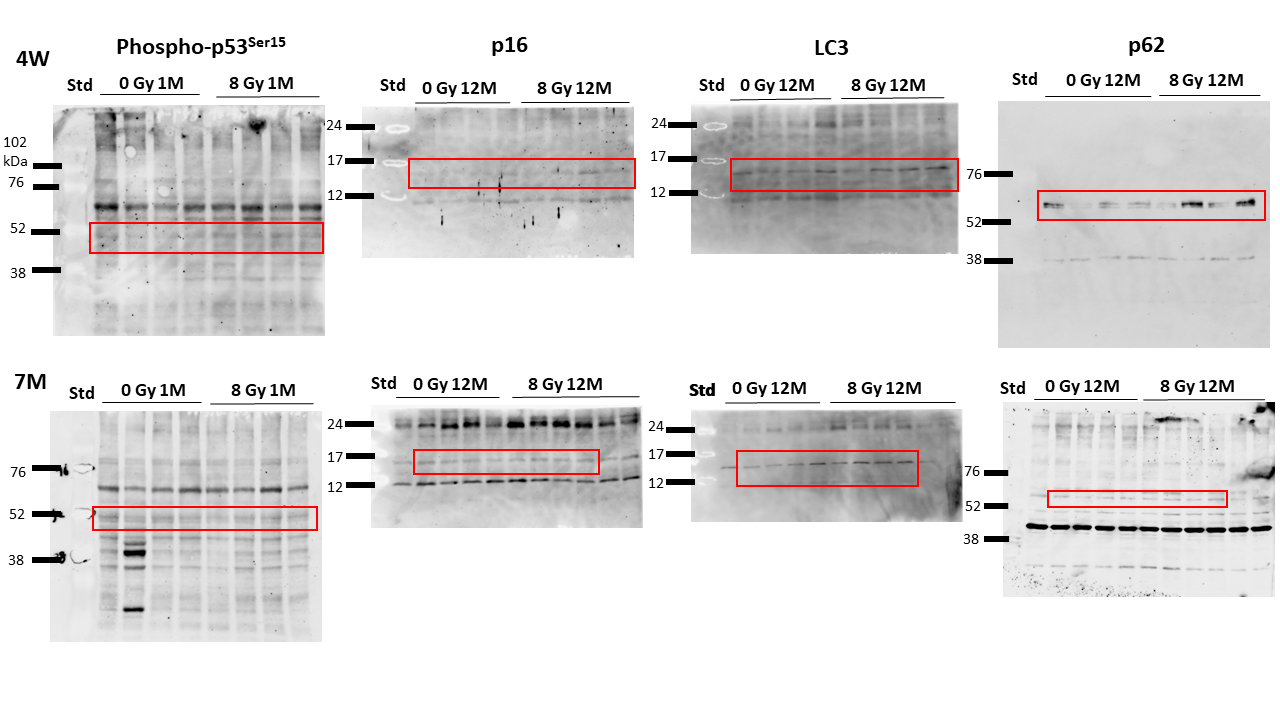


**Supplementary Fig. S3**

Full-length blots of phospho-p53^Ser15^, p16, LC3I and LC3II, and p62 in 4W and 7M rats in Fig.3.


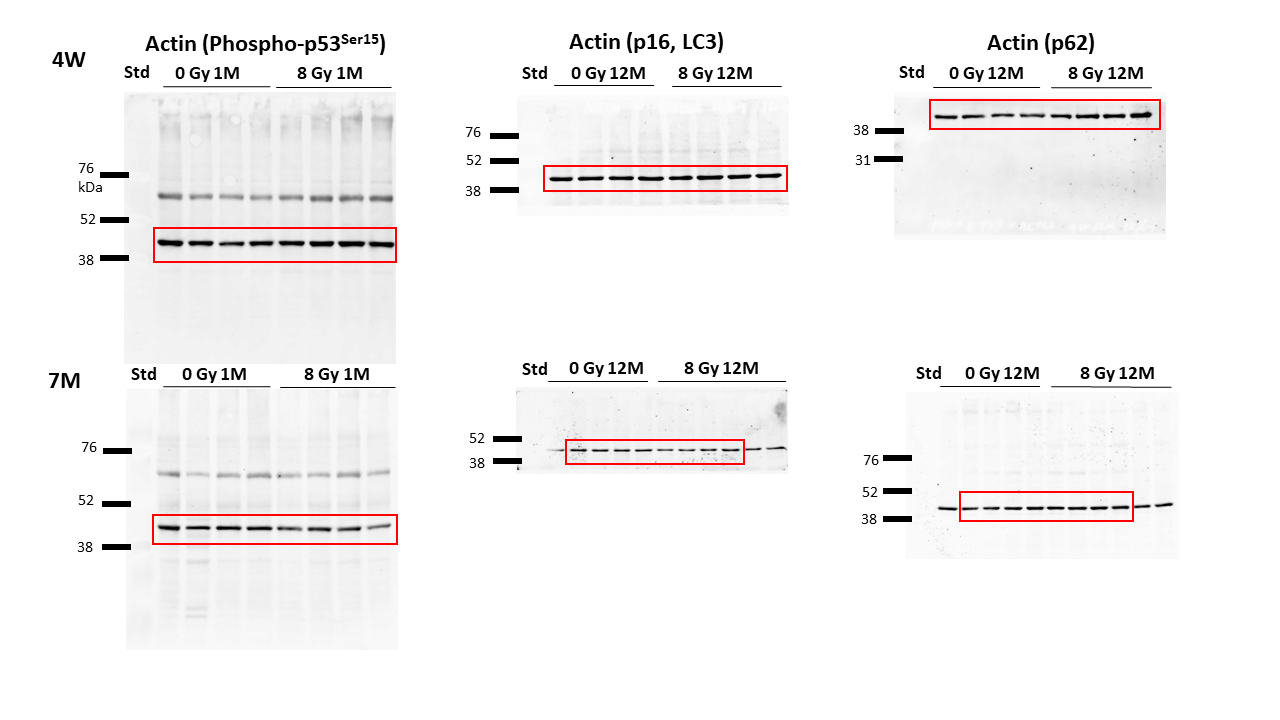


**Supplementary Fig. S4**

Full-length blots of actin in 4W and 7M rats in Fig.3.


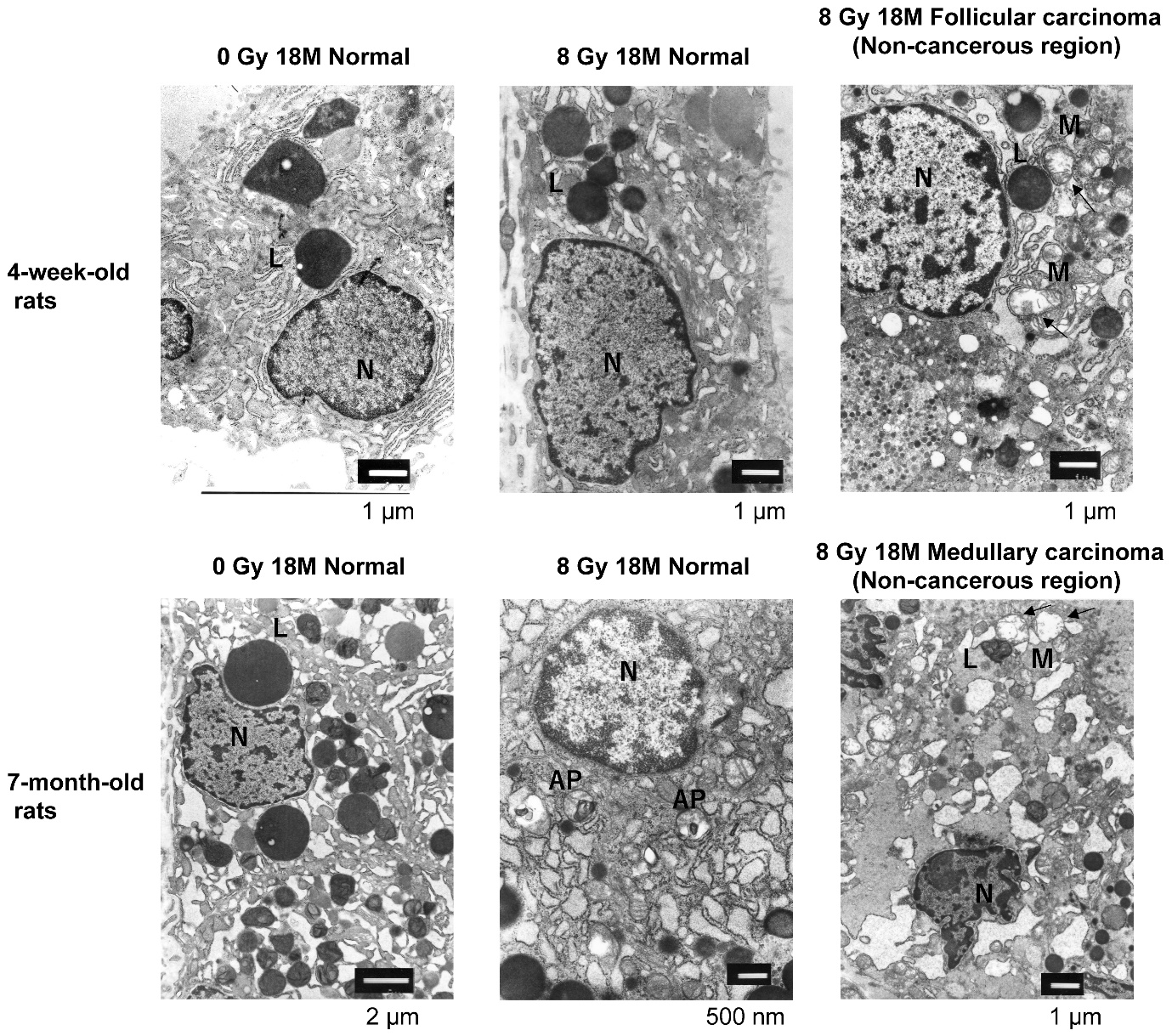


**Supplementary Fig. S5** Electron microscopy of nonirradiated normal thyroid tissue in 4W and 7M rats, noncancerous regions in follicular carcinoma in irradiated 4W rats, and medullary carcinoma in irradiated 7M rats

N, nuclear; L, lysosome; AP, autophagosome; M, mitochondria; arrow, mitochondrial swelling
